# Genomic and transcriptomic comparison of *Aspergillus oryzae* strains: a case study in soy sauce koji fermentation

**DOI:** 10.1007/s10295-018-2059-8

**Published:** 2018-07-05

**Authors:** Yiyi Zhong, Xi Lu, Lei Xing, Shiu Woon Allen Ho, Hoi Shan Kwan

**Affiliations:** 1 School of Life Sciences, The Chinese University of Hong Kong, Shatin, NT, Hong Kong SAR, China; 2 Food Research Centre, The Chinese University of Hong Kong, Shatin, NT, Hong Kong SAR, China; 3 Lee Kum Kee International Holdings Limited, Taipo, NT, Hong Kong SAR, China

**Keywords:** *Aspergillus oryzae*, Soy sauce, Koji fermentation, Genome, Transcriptome

## Abstract

The filamentous fungus *Aspergillus oryzae* is used in soy sauce koji making due to its high productivity of hydrolytic enzymes. In this study, we compared the genomes and transcriptomes of an industrial strain RD2 and a strain with decreased fermentation performance TS2, aiming to explain their phenotypic differences at the molecular level. Under the regulation of conidiation and fermentation conditions, the enhanced hydrolytic enzyme production and flavor precursor formation in RD2 described a complete expression profile necessary to maintain desirable fermentation performance. By contrast, central carbon metabolism was up-regulated in TS2 for fast growth, suggesting a conflicting relationship between mycelium growth and fermentation performance. Accumulation of mutations also lowered the fermentation performance of TS2. Our study has deepened the understanding of the metabolism and related regulatory mechanisms in desirable koji fermentation. A list of potential molecular markers identified here could facilitate targeted strain maintenance and improvement for better koji fermentation.

**Electronic supplementary material:**

The online version of this article (10.1007/s10295-018-2059-8) contains supplementary material, which is available to authorized users.

## Introduction

The filamentous fungus *Aspergillus oryzae*, also known as the koji mold, has been widely used in the production of traditional fermented food such as soy sauce, soybean paste and rice wine in China, Japan and other Asian countries for centuries. To make soy sauce, *A. oryzae* is cultured on a mixture of steamed soybeans and wheat flour. After a 3-day solid-state fermentation called koji fermentation, the resulting koji is then mixed with a brine solution for further submerged fermentation [11, 13]. Used as the starter of koji fermentation, *A. oryzae* has a prominent potential of secreting various hydrolytic enzymes to break down large molecules such as polysaccharides and proteins into smaller monosaccharides and amino acids [17, 26, 44]. These enzymes are fundamental and indispensable in the fermentation process, allowing prolonged degradation of biomass to energize subsequent fermentation and to supply metabolites that act as precursors for the characteristic flavor formation of soy sauce [9, 23].

Since the release of the first genome sequence of *A. oryzae* (RIB40 ATCC-42149) in 2005 [26], efforts have been made to understand the metabolism of this industrially important species. Comparative genomic studies have demonstrated that genes related to metabolism, especially hydrolytic processes, are overexpressed in *A. oryzae* compared to other aspergilli due to an expansion of these metabolic genes [17, 26]. Transcriptomic studies using microarray and RNA-Seq have revealed a high expression of metabolic genes in solid-state fermentation, thereby confirming the immense contribution of *A. oryzae* in fermentation as a source of enzymes and metabolites [28, 44]. For soy sauce, various *A. oryzae* strains have been isolated and selected for effective fermentation on soybeans, including the Japanese strain RIB326 [43] and the Chinese strain 3.042, which is equivalent to 3.951 [51]. These strains are common in producing a large number of hydrolytic enzymes, especially proteases, to adapt to the protein-rich environment during soybean fermentation, with acid protease possibly playing the most vital role in protein hydrolysis under the acidic environment of soy sauce fermentation [22, 48, 50]. High protease production can directly affect the amino acid content, and thus amino acid nitrogen, which is used to determine the quality grade of soy sauce in China [25].

Until now, the molecular mechanisms of *A. oryzae* metabolism and related regulations during soy sauce fermentation remain only partially understood, hindering targeted strain improvement and effective strain maintenance against industrial strain degeneration. For instance, although multiple hydrolases have been identified in soy sauce koji [22, 50], the transcriptional regulation of hydrolase production during koji fermentation remains unclear, not to mention the hydrolases that cannot be detected in proteomic approaches. To uncover the metabolism that is necessary to maintain koji fermentation performance, here we presented a case study of the comparison between a productive strain RD2 and strain TS2 that produced unfavorable soy sauce. Through integrated genomic and transcriptomic analysis, we identified a list of molecular markers and revealed the transcriptional regulatory mechanisms that are related to desirable koji fermentation.

## Materials and methods

### Strains and cultivation

Strains used in this study were obtained from a Chinese soy sauce factory: *A. oryzae* RD2 used in koji fermentation could produce good-quality soy sauce, whereas TS2 was isolated after repeated use of RD2, which produced soy sauce with a decreased amino acid yield and an unfavored flavor. The published genome of *A. oryzae* RIB40 [26] was downloaded from the *Aspergillus* Genome Database (AspGD) (http://www.aspergillusgenome.org/) (Version September 2016) and used as a reference.

RD2 and TS2 were cultivated on potato dextrose agar (PDA) plates at 30 °C for 3–4 days. Spores were collected and spread in autoclaved water to make an aqueous conidia suspension (ACS) (10^8^ spores/ml), which was stored at 4 °C until further use.

To produce koji, soaked soybeans were steamed, mixed with wheat flour, and inoculated with the prepared ACS. Koji fermentation was performed at 30 °C for about 72 h at ≥ 85% humidity. The process can be divided into three stages depending on the fermentation time and morphological changes: *stage I* mycelia expansion (ME), spans about 28 h, after which the surface of soybeans is covered with the white mycelia of *A. oryzae*; *stage II* early sporulation (ES), spans about 48 h, after which yellow-green spores appear on the white mycelia; and *stage III* mature sporulation (MS), spans about 72 h, after which the white mycelia is almost completely covered by spores, meaning that the koji is mature for subsequent fermentation.

### Strain characterization and enzyme activities

The mycelium growth diameter (in mm) of RD2 and TS2 on PDA plates was measured in triplicate in a daily basis. Growth rate was calculated as the mycelium growth diameter divided by the number of days (mm/d).

Protease and amylase activities at the ME, ES and MS stages of koji fermentation were determined in triplicate. To prepare crude enzyme solution, 5 g koji samples were mixed with 45 ml distilled water, then stirred at 40 °C for an hour. The filtered supernatant was centrifuged at 5000 g for 20 min. The protease activity was assayed by a modified method of Kum et al. [20], using 2% casein dissolved in pH 7.2 phosphate buffer (0.1 M Na_2_HPO_4_ plus 0.1 M KH_2_PO_4_) as substrate. A standard tyrosine solution was used to make a calibration curve for quantitative analysis. One unit of protease activity was defined as the quantity of protease that induced a change of 1 μg of tyrosine from 2% casein solution per minute. Amylase activity was assayed by a modified method of Fuwa [10], using 1% starch solution dissolved in pH 5.2 buffer (0.1 M citric acid plus 0.2 M Na_2_HPO_4_) as substrate and measurement at 580 nm absorbance. One unit of amylase activity was referred to the quantity of amylase that catalyzed the conversion of 1% soluble starch solution to 1 mg equivalent of maltose per minute.

pH of koji at the three stages was also determined from a well-mixed slurry of 5 g solution and 45 ml distilled water using a benchtop pH meter (Thermo Fisher Scientific).

### DNA extraction and ion torrent sequencing

Spores and mycelia of *A. oryzae* freshly grown on PDA plates at 30 °C for 3–4 days were collected and ground into fine powder with a mortar and pestle in liquid nitrogen. Genomic DNA was then extracted using the DNeasy Plant Mini Kit (Qiagen). Fragment DNA library construction was performed using the Ion Plus Fragment Library Kit (Thermo Fisher Scientific). About 100 ng of genomic DNA was enzymatically sheared by a Bioruptor (Diagenode) and ligated to Ion Torrent adapters. Fragments of 480 bp were selected by 2% agarose gel electrophoresis using the E-Gel iBase Power System and an E-Gel Safe Imager (Invitrogen, Thermo Fisher Scientific). Template for sequencing was prepared using the Ion OneTouch 2 System with the Ion PI Template OT2 400 Kit v2 (Thermo Fisher Scientific). Sequencing was performed on an Ion Torrent Personal Genome Machine (PGM 318 chip) using the Ion PGM Sequencing 400 Kit (Thermo Fisher Scientific).

### Genome mapping and variant calling

Quality of the raw DNA-Seq reads was first evaluated by FastQC (v0.11.4) [2]. Poor-quality reads (with a base quality < 20 in more than half of the bases) were filtered using the “fastq_quality_filter” command in the FASTX-Toolkit (http://hannonlab.cshl.edu/fastx_toolkit/). ShRiMP2 was then used to map the RD2 and TS2 genomes against RIB40, with the “− V” parameter adjusted from a previous method [7].

Uniquely mapped reads were used to perform single nucleotide variant (SNV) calling including single nucleotide polymorphisms (SNPs) and indel with the SAMtools “mpileup” command [21]. Only SNP-containing sites with at least three reads covering the position with an average quality ≥ 20 and indel-containing sites with at least five reads covering the position with an average quality ≥ 20 were extracted for further analysis. SNPeff [5] was then used to annotate the SNPs and indels with an SNPeff database created from RIB40 and custom parameters for fungal genomes.

### RNA extraction and Illumina HiSeq sequencing

Spores and mycelia were collected in duplicate from the three stages of koji fermentation and ground into powder with a mortar and pestle in liquid nitrogen. RNA was extracted using the RNeasy Micro Kit (Qiagen). RNA integrity was evaluated using gel electrophoresis and a NanoDrop Lite Spectrophotometer (Thermo Fisher Scientific). Transcriptomes were then sequenced on an Illumina HiSeq 2000 sequencing system at BGI Genomics.

### Transcriptome mapping and differential expression analysis

The raw RNA-Seq reads were first aligned to the *A. oryzae* RIB40 genome using TopHat. Cufflinks and Cuffdiff were then used to quantitatively normalize the expression levels to the number of fragments per kilobase of transcript per million mapped reads (FPKM) and to identify differentially expressed genes (DEGs) [42]. Genes with a fold change ≥ 1 and false discovery rate (FDR) < 0.05 were considered as significantly differentially expressed. The pheatmap package was used to generate heatmaps [18]. Functional analysis based on EuKaryotic Orthologous Groups (KOGs) was performed by subjecting DEGs to the WebMGA server (http://weizhong-lab.ucsd.edu/metagenomic-analysis) [47] using the kog functional annotation with an *e* value < 0.001. Pathway graphs were obtained from KEGG (http://www.genome.jp/kegg/).

### Validation of RNA-Seq data by real-time RT-PCR

RNA extracted was used in real-time RT-PCR to verify the RNA-Seq result. Genomic DNA-free cDNA was synthesized using iScript gDNA Clear cDNA Synthesis Kit (Bio-Rad). Real-time RT-PCR was performed on an ABI 7500 Fast Real-Time PCR System (applied biosystems) with iQ SYBR Green Supermix (Bio-Rad). Primers were designed in the exon regions of six selected genes (Table S1) and the expression of these genes was evaluated. The beta-tubulin gene (*btuA*) was used as a reference to normalize gene expression levels, and the relative transcriptional levels of the six genes were calculated (Fig. S1).

### Nucleotide sequence accession numbers

DNA-Seq and RNA-Seq data generated in this study are available in the sequence read archive (SRA) of NCBI under accession numbers SRP132444 and SRP132645, respectively.

## Results and discussion

### Strain growth and enzyme activities

**Fig. 1:**
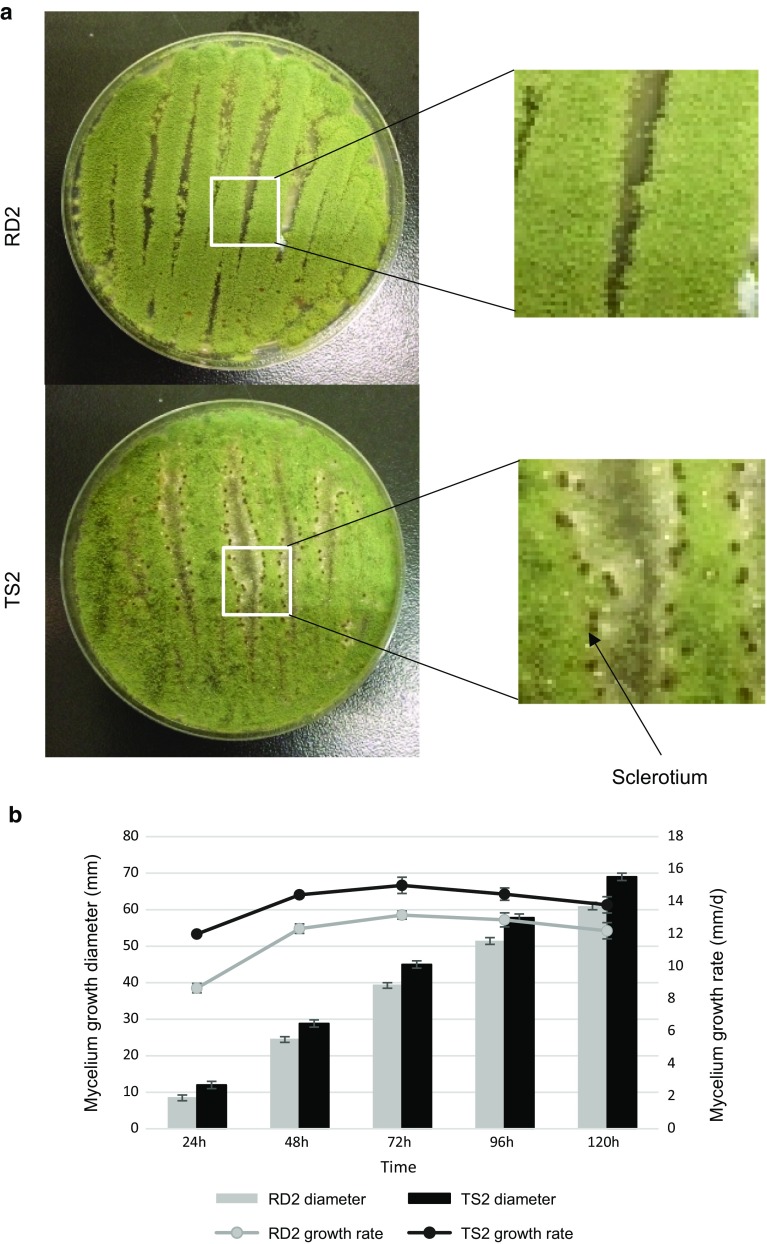
Growth phenotypes of RD2 and TS2 on PDA plates. **a** RD2 and TS2 grown after 72 h. The arrow indicates a sclerotium (dark spot) produced by TS2. **b** Mycelium growth diameter and growth rate of RD2 and TS2. Data are presented as mean ± standard deviation. Significant difference was detected between RD2 and TS2 in all days (*P *< 0.01, Student’s *t* test)

**Fig. 2:**
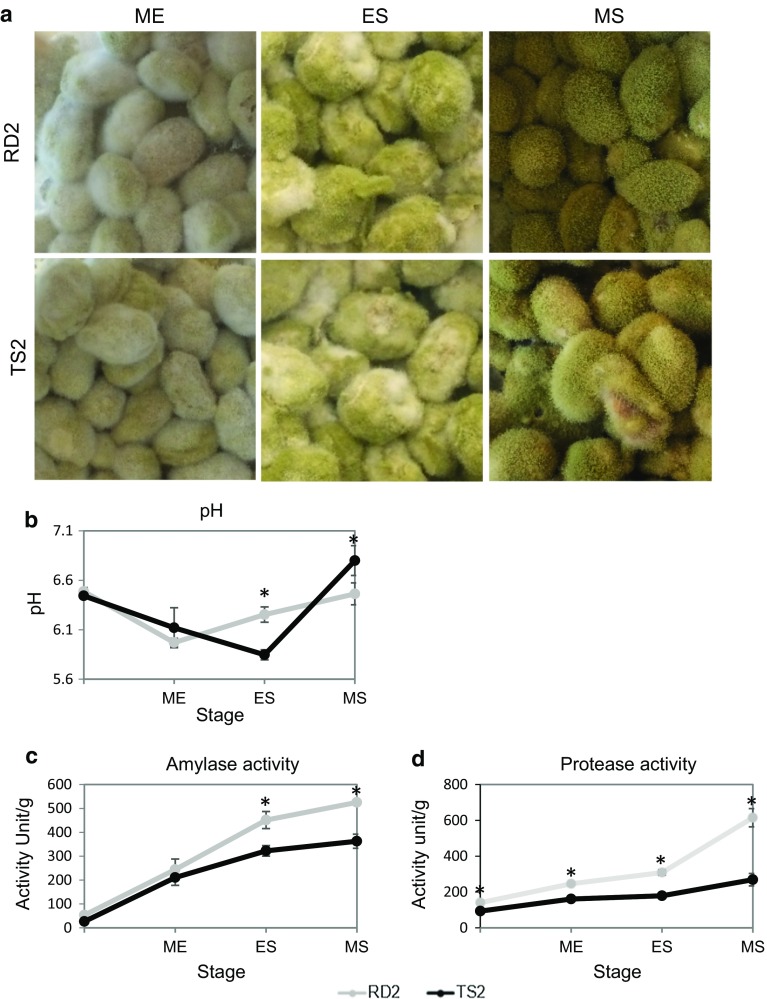
Phenotypes of RD2 and TS2 during koji fermentation in three fermentation stages: growth (**a**), pH (**b**), amylolytic activities (**c**), and proteolytic activities (**d**). *ME* mycelium expansion, *ES* early sporulation, *MS* mature sporulation. Data are presented as mean ± standard deviation. *P < 0.05 (Student’s *t* test)

RD2 and TS2 exhibited different phenotypes on PDA plates. TS2 possessed a significantly higher mycelium growth rate (13.9 ± 1.2 mm/d) compared to RD2 (11.8 ± 1.8 mm/d) (Fig. 1b), showing a growth advantage of TS2 over RD2 and TS2 began conidiation at 32 h, which is 4 h ahead of RD2. However, RD2 displayed complete conidiation with yellow-green spores, whereas conidiation in TS2 was decreased, especially at the edge of colonies, with asporogenous mycelium left (white hyphae) and sclerotia formed (dark spots) (Fig. 1a). Sclerotia formation has been reported in *A. oryzae* as a result of aberrant branching and intertwining of mycelia [15]. Normally, after gemination of spores, *A. oryzae* acquires nutrients for mycelium or vegetative growth to penetrate the substrate, aerial hyphae then develop into conidiophores to perform conidiation [40]. Therefore, the decreased conidiation observed in TS2 is probably because of the fact that less aerial hyphae could successfully develop into conidiophores. Also, TS2 showed a faster growth than RD2 in the ME, ES, MS stages of koji fermentation, as it accomplished each stage in a shorter time, but with decreased conidiation especially in the MS stage (Fig. 2a).

The pattern of pH change differed in koji fermented by the two strains (Fig. 2b). In RD2 koji, pH first decreased from 6.5 to 6.0 in the ME stage, then increased gradually back to 6.5; while in TS2 koji, pH first declined from 6.5 to 5.8 in the ES stage, and then rose to 6.8. The pH change in RD2 koji is similar to that reported in previous study [22]. Nevertheless, the abnormal pH change from the ME to MS stage in TS2 koji might indicate different metabolic activities of TS2.

The amylase and protease activities of the two strains during koji fermentation were assayed and compared (Fig. 2c, d). Both strains displayed increasing enzyme activities along fermentation due to the accumulation of enzymes. However, RD2 showed a significantly higher protease activity than TS2, especially during the transition from the ES to MS stage, after which the protease activity of RD2 was threefold higher than that of TS2 (Fig. 2c). The lower protease activity in TS2 koji could directly result in a decreased amino acid yield and thus soy sauce with a poor quality. RD2 also displayed a significantly higher amylase activity than TS2 in the ES and MS stages (Fig. 2d), showing a stronger ability to utilize amylolytic carbohydrates in late koji fermentation. Noticeably, complete conidiation occurred synchronously with high enzyme activities in RD2, suggesting a correlation between conidiation and enzyme production. Indeed, te Biesebeke et al. [40] have proven that the production of amylases and proteases is positively related to the number of penetrating hyphae, which is the main apparatus to produce hydrolytic enzymes. Here, more spores in RD2 could geminate to grow more penetrating hyphae, which facilitated enzyme production. Thus, conidiation could reflect the level of enzyme production.

### General genomic statistics and variant calling

DNA sequences of both strains were obtained by whole-genome sequencing using the Ion Torrent platform. General statistics of the genomes is summarized in Table 1. Aligning the reads to the reference RIB40 genome showed that high-quality reads from both RD2 and TS2 covered over 95% of the reference genome with an average depth of ~ 7*x*. Using SHRiMP2, 96.3% of the RD2 reads and 95.2% of the TS2 reads were successfully mapped to the RIB40 genome. The internal transcribed spacer (ITS) and calmodulin sequences were recommended for the identification of aspergilli [36]. Sequences of both gene regions of TS2 showed 100% identity to those of RD2 and RIB40, excluding the possibility of contamination. Using the RD2 genome as reference, 858 SNPs and 50 indels were identified in TS2. The majority of SNVs (64.3% of SNPs, 74% of indels) occurred in the intergenic region. Excluding synonymous SNPs and mutation occurred in introns, 81 SNPs were predicted to cause 79 missense and two nonsense effects, and nine indels were predicted to cause frameshift effects, which could directly affect the structure and function of 66 genes (Table S3).

**Table 1:** General statistics of RD2 and TS2 genomes

	RD2	TS2
Total number of bases	318.08 Mbp	298.14 Mbp
Total number of reads	1,836,901	1,706,630
Number of Q20 bases^a^	296.73 Mbp	279.25 Mbp
Number of Q20 reads^b^	1,645,434	1,538,563
Filtered reads^c^	191,467	168,067
Mean read length	172 bp	173 bp
Longest read length	366 bp	321 bp
GC content	47%	46%

^a^Bases with quality ≥ 20 are counted

^b^Reads with a base quality ≥ 20 in more than half of the bases are counted

^c^Reads with a base quality < 20 in more than half of the bases are filtered

It is noteworthy that SNVs of TS2 were identified in genes related to hydrolysis of carbohydrates and amino acids. For instance, missense mutations were identified in beta-glucosidase (*AO090001000266*), alpha-glucanase (*AO090113000091*) and carboxypeptidase C (*AO090023000382*); and a frameshift mutation was identified in tripeptidyl-peptidase (*AO090701000907*). These SNVs could result in malfunctional glycolytic and proteolytic enzymes and lower enzyme activities in TS2. SNPs found in two transcriptional factors (TFs) with predicted roles in carbohydrate metabolism regulation (*AO090005001296*, *AO090103000131*) could also influence the glycolytic activity of TS2. Interestingly, three missense mutations were identified in translation initiation factor 5A (*elF*-*5A*), which is specifically expressed in the penetrating hyphae to facilitate polypeptide synthesis during translation [29]. Since penetrating hyphae is the main apparatus to secrete hydrolytic enzymes [40], we speculate that TS2 is inferior in protein synthesis and thus enzyme secretion. Two mutated genes related to arginine metabolism are highlighted here: transmembrane arginine transporter (*AO090023000039*) and TF involved in arginine biosynthesis (*AO090701000493*). Arginine is a critical amino acid required for mycelium growth and conidiation, where a higher concentration is required for conidiation than mycelium growth [1]. The mutated arginine transporter and biosynthesis regulator could lower the concentration of arginine produced, leading to the conidiation defects in TS2.

TS2 also accumulated mutations in the metabolism of amino acids, fatty acids and isoprenoids, which can be precursors of flavor compounds. For instance, SNPs identified in cysteine synthase (*AO090011000336*) and glutathione S-transferase (*AO090038000465*) could hinder the enzymatic mediation of cysteine production, which would impair the formation of sulfur-containing flavors [38]. Indeed, protein metabolism and amino acid profiles have been suggested to play pivotal role in the determination of soy sauce flavor [23, 49]. SNPs found in the potential regulator of amino acid biosynthesis (*AO090020000331*) and amino acid transporters (*AO090026000734*, *AO090003000832*) could therefore have prolonged negative effect on soy sauce flavor formation. Mutated genes related to fatty acid metabolism included lipase 2 precursor (*AO090103000172*), fatty acyl-CoA synthase (*AO090001000249*), and fatty acid synthase (*AO090010000108*). As fatty acids are precursors of predominant aroma in soy sauce [9], SNVs in lipid hydrolysis and fatty acid biosynthesis also explain the defects in flavor formation in TS2. Three missense mutations found in geranylgeranyl pyrophosphate synthase (*AO090009000093*) involved in the biosynthesis of isoprenoids may represent another evidence. Isoprenoids are aroma precursors identified in fermented soy sauce and soybean paste [13]. They can undergo intricate rearrangement and isomerization under acidic conditions to yield odor-active compounds such as methyl butanone derivatives, which have been detected in both koji and soy sauce [9, 38, 49].

### Transcriptome comparison during koji fermentation

**Fig. 3:**
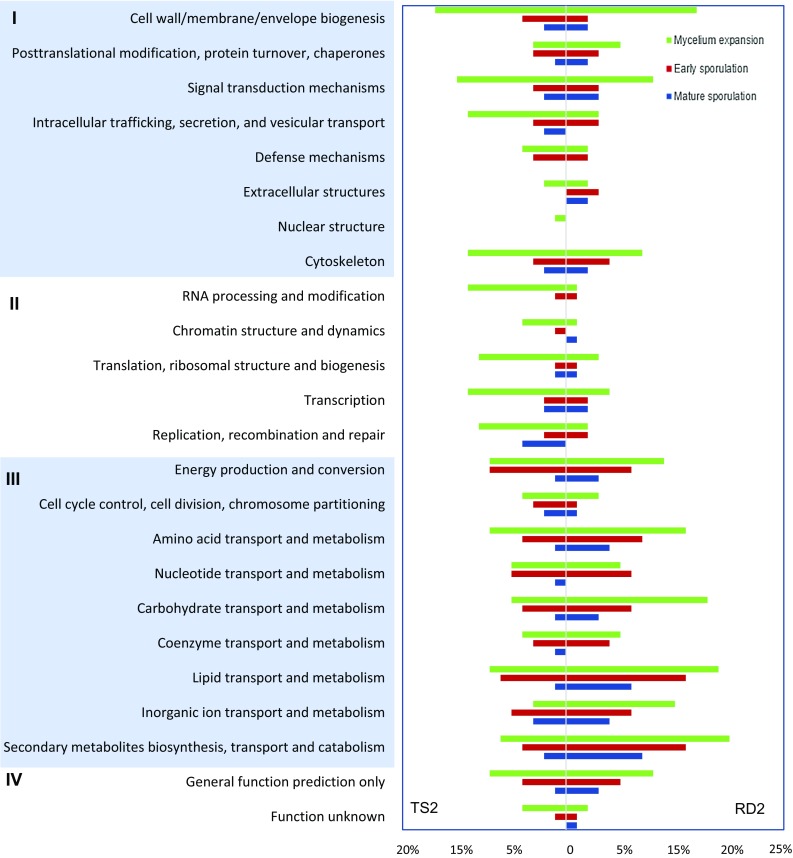
EuKaryotic Orthologous Groups (KOG) analysis. Main categories: *I* cellular processes and signaling, *II* information storage and processing, *III* metabolism, *IV* poorly characterized

To investigate the transcriptomic differences of RD2 and TS2 during koji fermentation, RNA extracted from three fermentation stages were subject to high-throughput Illumina sequencing. An average of 28,659,093 reads were obtained for each of the 12 samples (two strains in three stages, with duplicates). Over 90% raw reads could be mapped to the RIB40 genome with a tolerance of two mismatches (Table S1). A total of 1796, 1008 and 472 DEGs were identified in the ME, ES and MS stages, respectively. Functional analysis based on KOG revealed that DEGs up-regulated in RD2 were enriched in metabolism, especially transport and metabolism of carbohydrates, amino acids, lipids, and secondary metabolites; whereas TS2 possessed more up-regulated genes in information storage and processing, including DNA replication, transcription and translation (Fig. 3). Since RD2 yields better soy sauce when used in the koji fermentation, the differential expression profiles indicate key metabolism and transcriptional regulation that maintain the desirable koji fermentation performance. In addition, the number of DEGs decreased over time, indicating a stronger cellular response in early fermentation stage when nutrients were sufficient for growth and development. To verify the transcriptome data, six genes were selected for real-time RT-PCR (Table S2). The trends of up- and down-regulation of these genes were confirmed to be consistent with the RNA-Seq results (Fig. S1).

### Regulation of glycolytic and proteolytic enzyme production

**Fig. 4:**
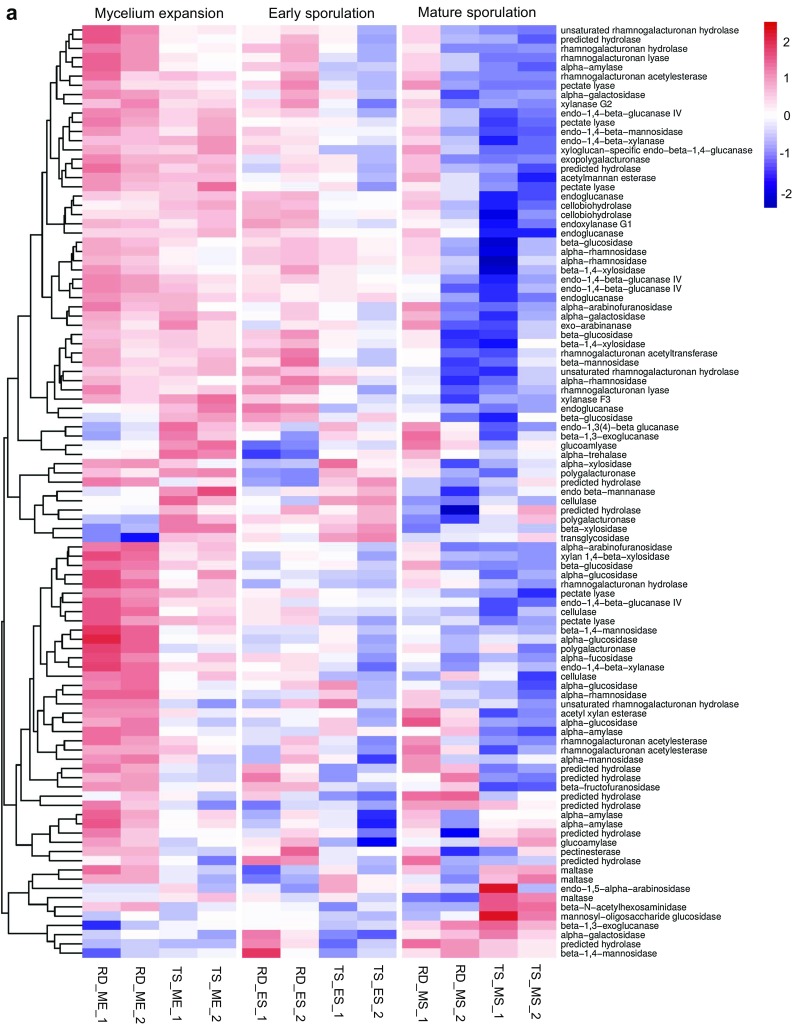

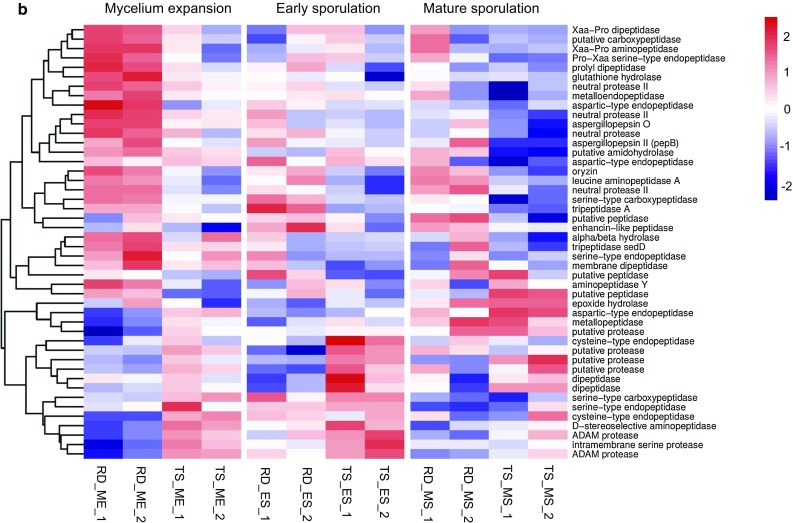
Heatmaps from hierarchical clustering of differentially expressed genes encoding degrading enzymes of carbohydrates (**a**) and proteins (**b**)

The ability to produce hydrolytic enzymes by *A. oryzae* is of great concern when considering the performance of koji fermentation. To investigate the expression of glycolytic and proteolytic enzymes during koji fermentation, we analyzed the identified DEGs based on the carbohydrate-active enzyme database (http://www.cazy.org/) [24] and MEROPS peptidase database (https://www.ebi.ac.uk/merops) [35]. Results revealed that 101 and 46 DEGs were involved in the hydrolysis of carbohydrates and proteins, respectively (Fig. 4), including both well-curated and in silico predicted hydrolytic enzymes. In general, RD2 possessed more up-regulated genes encoding hydrolytic enzymes than TS2, supporting the higher enzyme activities observed in RD2. We thereby provided a completed expression profile of important hydrolytic enzymes that are necessary to maintain koji fermentation performance.

Besides the well-known amylolytic enzymes that are highly expressed in soy sauce-producing strains [22, 28, 43], various cellulolytic, xylanolytic and pectinolytic enzymes were also expressed in significantly higher levels in RD2 in the ME and ES stages (Fig. 4a). Among these, alpha-galactosidases, up-regulated in RD2 in all three stages, can effectively degrade the soybean sugars—raffinose and stachyose. Since these enzymes require corresponding carbohydrates for inducement [14], our results suggest a better hyphal penetration of RD2 into soybeans, which contain the most plant polysaccharides including cellulose, pectin, raffinose, and stachyose within the substrates [8]. A network of TFs has been reported to regulate the expression of these enzymes [19]. Here, three up-regulated TFs in RD2 might be highly related: *AO090020000656*, a homolog of *clrA* in *A. nidulans* that functions as the activator of cellulases and xylanases [6]; *manR*, an activator of beta-glucosidases and alpha-galactosidases in the degradation of cellulose, hemicellulose, raffinose, and stachyose [32]; and *AO090005000121*, a homolog of *rhaR* in *A. niger* that acts as the activator of rhamnogalacturonan hydrolase and pectin lyases involved in pectin degradation [12].

By contrast, few plant polysaccharide-degrading enzymes were induced in TS2, since there was no up-regulation of the aforementioned regulators and the hyphal penetration was limited. However, there was an up-regulation of glucose-release enzymes including maltase and trehalase in the ES and MS stages of TS2, suggesting that this strain exhaustedly exploited preferred carbon source maltose for proliferation. In fact, it is less energy-intensive and more cost-efficient to directly use preferred carbon source than secrete plant polysaccharide-degrading enzymes for alternative carbon sources [19]. This potential enables a fast growth of TS2 as observed in Figs. 1a, 2a. On the other hand, active metabolism of various polysaccharides in RD2 (Fig. S2) ensures a simple sugar reserve for subsequent fermentation and facilitates the utilization of proteins as plant polysaccharide-degrading enzymes are coordinately responsible for the degradation of plant cell walls and the solubilization of proteins [17, 19].

Production of protease is a major concern in soy sauce koji fermentation since it directly relates to the amino acid yield and thus product quality. Here, RD2 possessed highly up-regulated peptidases including acid proteases aspergillopepsins, neutral proteases and alkaline protease oryzin in all three stages (Fig. 4b). This result is consistent with the high protease yield reported in other soy sauce-producing strains [43, 50]. The largest fold change (up to 18-fold) was observed in aspergillopepsin II (*AO090023000393*), which was barely expressed by TS2, especially in the MS stage. Other acid proteases including serine-type carboxypeptidase (*AO090012000706*), tripeptidase A (*tppA*) and aspergillopepsin O (*pepO*) were also expressed in extremely low levels in TS2 in the MS stage. Here, pH could act as a regulator because *pacC* was expressed at an about fivefold higher level in TS2 in the MS stage, at which the pH of koji experienced a dramatic increase (Fig. 2b). *PacC* is active in alkaline conditions and can repress acid-expressed genes and activate alkaline-expressed genes in *A. nidulans*, or function only as a repressor of acid-expressed genes in *A. niger* or *Saccharomyces cerevisiae* [34]. It can specifically bind to its target genes with the binding sequence of 5′-GCCARG-3′, which is present in the promoter region of *AO090012000706*, *tppA*, *pepO* at the –242, –290, –308 positions, respectively. *A. oryzae* grows in a slightly acidic condition during koji fermentation, but it may suffer from stress in response to rapid alkalization [34], as shown in TS2 (Fig. 2b). Therefore, highly induced *pacC* in TS2 probably represses the expression of acidic proteases in late fermentation stage. A higher production of acidic proteases has been reported to be advantageous in substrate utilization and product quality improvement [48, 50]. Thus, it is possible to improve koji fermentation performance by monitoring pH or manipulating pH-related TFs like *pacC*. It is noteworthy that alkaline proteases leucine aminopeptidase and oryzin, and neutral proteases I and II were also expressed in extremely low levels in TS2, especially in the MS stage, leading to lower protease activity in late fermentation stage. te Biesebeke et al. has suggested the induction of *pacC* on oryzin and neutral proteases [41]. However, no inductive effect was observed upon the up-regulation of *pacC* in TS2 here.

Some other regulatory mechanisms may also be responsible for the overall enhanced protease production in RD2. As aforementioned, conidiation can be linked to enzyme production. In fact, protease production of *A. oryzae* has been proven to be closely related to conidiation [3]. A possible explanation is that some TFs positively regulating conidiation also play inductive roles in protease production [31, 39]. One example is the sporulation activator *flbC*, as *flbC*-disrupted mutant shows significantly decreased expression of acid protease *pepO* and glucoamylase *glaB* [39]. Here, we found that three regulators of conidiation, *flbC*, *atfB* and *AO090003001259*, were up-regulated in RD2 in one or more stages. Although the exact mechanism is unknown, these regulators likely contribute to the increased protease production in RD2. In addition, the better hyphal penetration due to up-regulated plant polysaccharide-degrading enzymes in RD2 may allow better exposure to proteins inside soybeans, which can also contribute to protease production [3].

### Regulation of central carbon metabolism on koji fermentation performance

**Fig. 5:**
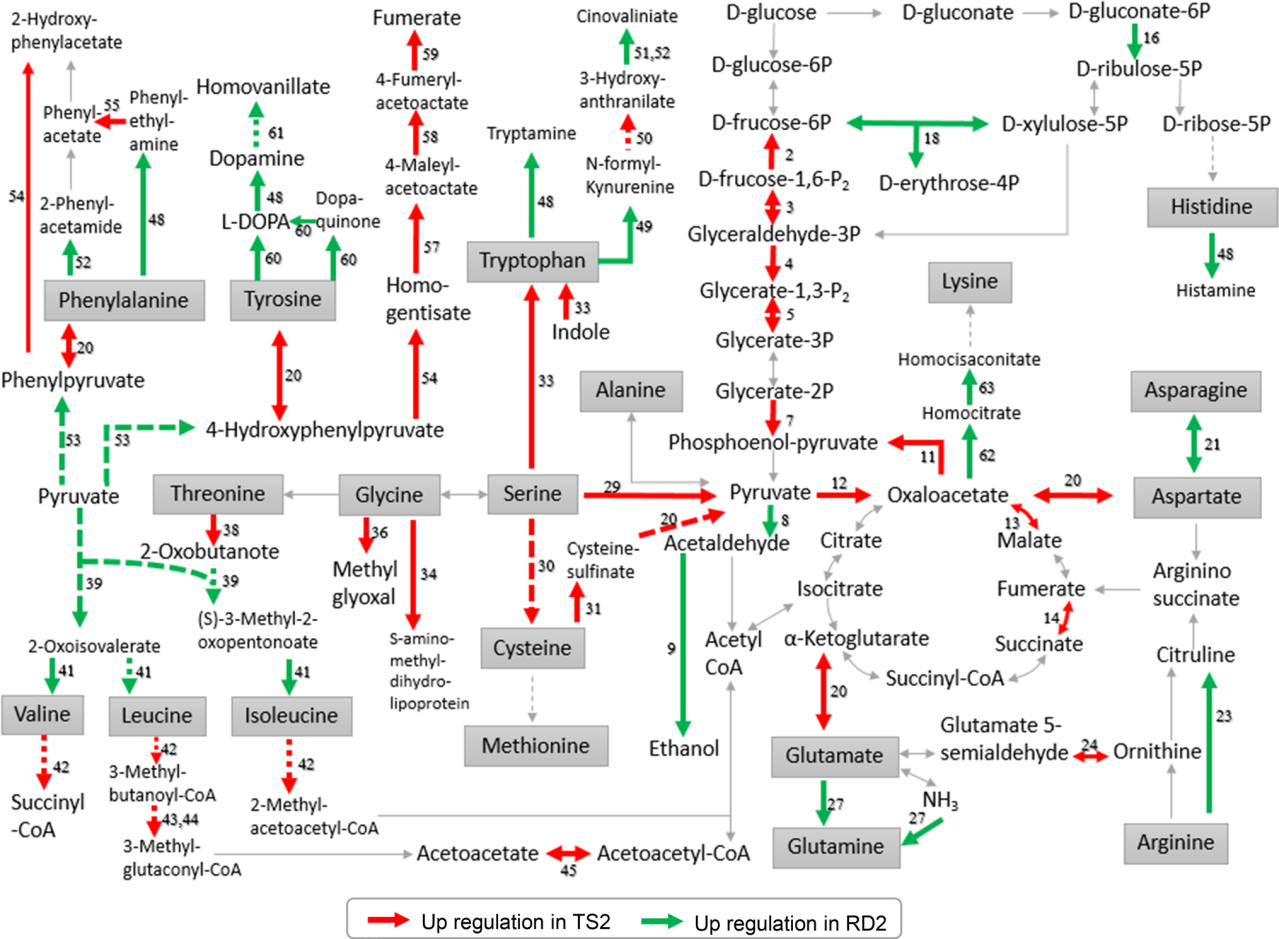
Simplified KEGG pathways showing differentially expressed genes in the early sporulation stage that were mapped on the central carbon metabolism and related amino acid metabolism. Red and green arrows indicate up-regulated reactions in TS2 and RD2, respectively; dashed arrows indicate more than one step of reaction involved. DEG numbers correspond to the enzymes listed in Table 2. *P* phosphate, *l-DOPA* 3,4-dihydroxy-l-phenylalanine

One of the most striking findings of this study is the distinct gene expression profiles of central carbon metabolism between the two strains (Fig. 5, S3). In the ES stage, glycolysis and TCA cycle in TS2 were highly induced (Fig. 5, Table 2). However, in RD2, pentose phosphate pathway was induced. These results indicate different fates of glucose in the two strains.

**Table 2:** Expression fold change of differentially expressed genes (DEGs) involved in central carbon metabolism and related metabolism

Number	Gene ID	EC number	Gene function	Log_2_ (fold change)^a^		
				ME	ES	MS
1	*pfkA*	2.7.1.11	6-Phosphofructokinase	1.09	− 0.36	− 0.56
2	*fbpA*	3.1.3.11	Fructose-1,6-bisphosphatase	− 0.06	1.58	− 0.68
3	*fbaA*	4.1.2.13	Fructose-1,6-bisphosphate aldolase	0.05	1.43	− 0.25
4	*gpdA*	1.2.1.12	Glyceraldehyde-3-phosphate dehydrogenase	− 0.13	1.86	− 0.74
5	*pgkA*	2.7.2.3	3-Phosphoglycerate kinase	0.09	2.08	− 0.86
6	*gpmA*	5.4.2.12	Phosphoglyceromutase	1.16	0.54	− 0.23
7	*enoA*	4.2.1.11	Enolase	0.13	1.60	− 0.24
8	*AO090003000661*	4.1.1.1	Pyruvate decarboxylase	− 0.31	− 1.64	− 1.50
9	*AO090003000261*	1.1.1.1	Alcohol dehydrogenase	− 2.45	− 1.29	− 2.33
10	*AO090023000467*	1.2.1.3	Aldehyde dehydrogenase	− 1.03	− 0.85	0.14
10	*AO090026000741*	1.2.1.5	Aldehyde dehydrogenase	− 1.13	0.19	− 0.80
11	*AO090003000174*	4.1.1.49	Phosphoenolpyruvate carboxykinase	1.90	4.62	− 0.96
12	*AO090023000801*	6.4.1.1	Pyruvate carboxylase	− 0.16	1.39	− 0.51
13	*mdhA*	1.1.1.37	Malate dehydrogenase	0.21	1.55	0.19
14	*sdhB*	1.3.5.1	Succinate dehydrogenase	− 0.15	1.38	− 0.43
14	*sdhC*	1.3.5.1	Succinate dehydrogenase	− 0.23	1.06	− 0.33
15	*AO090003001003*	2.7.1.12	Gluconokinase	1.24	− 0.41	− 0.72
16	*AO090003000121*	1.1.1.44	6-Phosphogluconate dehydrogenase	0.11	− 1.52	− 0.09
17	*AO090026000544*	5.3.1.6	Ribose-5-phosphate isomerase A	− 1.51	− 0.26	− 0.36
18	*AO090012000526*	2.2.1.1	Transketolase	− 0.02	− 1.19	0.12
19	*AO090003000625*	4.1.2.9	Fructose-6-phosphate phosphoketolase	1.81	0.93	− 1.85
20	*AO090003001171*	2.6.1.1	Aspartate transaminase	− 0.10	1.25	− 1.23
21	*AO090120000180*	6.3.5.4	Asparagine synthase	− 1.21	− 1.38	0.53
22	*AO090023000395*	6.3.4.5	Argininosuccinate synthase	− 1.35	− 0.71	− 0.78
23	*AO090138000171*	1.14.13.39	Nitric oxide synthase	− 1.09	− 1.46	0.22
24	*AO090023000546*	2.6.1.13	Ornithine-oxo-acid transaminase	− 0.03	1.82	0.60
25	*AO090001000549*	1.2.1.88	1-Pyrroline-5-carboxylate dehydrogenase	− 2.42	0.21	− 0.50
26	*AO090005000539*	4.1.1.15	Glutamate decarboxylase	1.16	− 0.32	− 0.52
27	*AO090011000308*	6.3.1.2	Glutamine synthetase	2.69	− 2.98	0.86
28	*gtaA*	3.5.1.2	Glutaminase	− 0.16	− 0.19	− 1.54
29	*AO090023000790*	4.3.1.17	Serine/threonine amonialyase	0.92	3.19	− 0.17
30	*AO090102000630*	2.5.1.47	Cysteine synthase A	− 0.10	1.29	− 0.83
31	*AO090020000659*	1.13.11.20	Cysteine dioxygenase	− 2.74	3.25	− 2.08
32	*AO090038000357*	2.5.1.6	*S*-Adenosylmethionine synthetase	− 0.02	− 0.11	1.05
33	*AO090023000874*	4.2.1.20	Tryptophan synthase	− 1.11	1.22	0.46
34	*AO090011000351*	1.4.4.2	Glycine dehydrogenase	0.41	1.62	0.21
35	*AO090005000497*	4.1.2.48	Threonine aldolase	1.46	0.25	1.31
36	*AO090005000103*	1.4.3.21	Primary amine oxidase	− 2.21	1.25	0.15
37	*AO090005000038*	1.5.3.1	Sarcocine oxidase	− 1.15	− 0.76	− 0.70
38	*AO090023000790*	4.3.1.19	Threonine dehydratase	0.92	3.19	− 0.17
39	*AO090166000076*	2.2.1.6	Acetolactate synthase	1.55	− 1.07	1.32
40	*AO090009000414*	4.2.1.9	Dihydroxy acid dehydratase	− 1.43	− 0.18	− 1.47
41	*AO090005000936*	2.6.1.42	Branched-chain amino acid transaminase	− 0.56	− 1.79	− 0.81
41	*AO090011000044*	2.6.1.42	Branched-chain amino acid transaminase	− 0.32	0.44	− 1.31
42	*AO090001000555*	1.2.4.4	Branched-chain amino acid alpha-keto acid dehydrogenase	− 1.58	2.40	− 1.03
43	*ivdA*	1.3.8.4	Isovaleryl-CoA dehydrogenase	− 1.58	1.66	− 0.35
44	*AO090020000492*	6.4.1.4	3-Methylcrotonyl-coA carboxylase	− 1.35	1.35	− 0.12
45	*AO090009000195*	2.8.3.5	3-Oxoacid CoA-transferase	− 1.37	1.12	− 1.28
46	*AO090103000406*	2.3.1.9	Acetyl-CoA acetyltransferase	− 1.43	0.87	− 1.00
47	*AO090023000518*	1.1.1.31	3-Hydroxyisobutyrate dehydrogenase	− 0.72	− 0.36	− 1.08
48	*AO090038000057*	4.1.1.28	Aromatic amino acid decarboxylase	− 1.23	− 1.07	0.35
49	*AO090038000578*	1.13.11.52	Indoleamine 2,3-dioxygenase	− 1.38	− 1.97	0.06
50	*AO090003001247*	3.7.1.3	Kynureninase	− 0.79	3.97	− 1.96
51	*catA*	1.11.1.6	Catalase	0.53	− 1.23	− 0.61
51	*catB*	1.11.1.6	Catalase	− 1.49	− 0.43	− 0.90
52	*AO090010000722*	1.11.1.21	Catalase peroxidase	0.50	− 1.90	2.20
53	*AO090005000086*	2.5.1.54	3-Deoxy-7-phosphoheptulonate synthase	− 0.15	− 1.15	− 0.77
54	*AO090003000208*	1.13.11.27	4-Hydroxyphenylpyruvate dioxygenase	− 1.35	6.25	− 2.99
55	*AO090005000103*	1.4.3.21	Copper amine oxidase	− 2.21	1.25	0.15
56	*AO090003001361*	1.14.14.54	Phenylacetate 2-hydroxylase	− 2.25	− 0.33	− 1.36
57	*AO090003000210*	1.13.1.15	Homogentisate 1,2-dioxygenase	− 1.47	4.26	− 2.00
58	*AO090003000212*	5.2.1.2	Maleylacetoacetate isomerase	− 0.49	3.00	− 1.48
59	*AO090003000211*	3.7.1.2	Fumarylacetoacetase	− 1.18	3.22	− 0.98
60	*melO*	1.14.18.1	Tyrosinase	− 1.63	− 2.82	− 1.41
61	*AO090701000285*	2.1.1.6	Catechol-*O*-methyltransferase	0.22	− 1.83	0.73
62	*AO090003001165*	2.3.3.14	Homocitrate synthase	0.56	− 1.19	0.01
63	*AO090001000484*	4.2.1.-	Homoaconitase	0.07	− 1.10	− 0.26
64	*AO090001000233*	1.5.1.10	Saccharopine dehydrogenase	1.53	− 0.62	− 0.62
65	*AO090012000450*	2.6.1.9	Histidinol phosphate aminotransferase	0.05	0.07	1.05

^a^Log_2_ (fold change) < − 1 indicates the up-regulation in RD2, log_2_ (fold change) > 1 indicates the up-regulation in TS2

In TS2, glucose is first channeled to phosphoenolpyruvate through up-regulated *fpbA*, *gpdA*, *pgkA*, and *enoA*, thereby generating more pyruvate, which then entails a large cascade of TCA cycle reactions involving up-regulated *AO090023000801*, *mdhA* and *sdhB*, *sdhC*. The up-regulated TCA cycle in TS2 possibly supports the pH decrease in the ES stage (Fig. 2b) because acids produced via the TCA cycle, such as succinate and citrate, are the major reason of acidification during koji fermentation [33]. Apart from the larger amount of pyruvate generated by glycolysis, the up-regulated aspartate transaminase and serine/threonine ammonia-lyase involved in the catabolism of glutamate, aspartate, cysteine, tyrosine, and serine to pyruvate and TCA metabolites can also feed the TCA cycle in TS2. Thus, more ATPs can be generated via the TCA cycle in TS2, providing sufficient energy to support the vegetative growth of multicellular microorganisms [4], which explains the fast growth of TS2. By contrast, reduced expression of genes involved in glycolysis and TCA cycle and increased expression of those involved in hydrolytic enzyme production were observed in RD2. A low expression of glucose catabolic genes could release catabolite repression, contributing to the elevated level of hydrolytic enzyme production [28]. Therefore, glycolysis and TCA cycle may play a contradictive role in hydrolytic enzyme production, and highly induced glycolysis and TCA cycle in TS2 is unfavorable during koji fermentation.

In RD2, up-regulated *AO090003000121* and *AO090012000526* direct the glucose metabolism to the pentose phosphate pathway (Fig. 5). The pentose phosphate pathway generates xylulose-5-phosphate, ribulose-5-phosphate and ribose-5-phosphate, which are precursors of characteristic flavor compounds in soy sauce such as 4-hydroxy-2(or 5)-ethyl-5(or 2)-methyl-3(2H)-furanone (HEMF), guanosine-5′-monophosphate (GMP) and inosine-5′-monophosphate (IMP) [38]. It is evident that the metabolic fate of glucose in RD2 is channeled to flavor precursor accumulation for better koji fermentation. Besides, genes involved in umami-flavored glutamate biosynthesis, including glutaminase and glutamate dehydrogenase, were up-regulated in the late fermentation stage. Branched-chain amino acid transaminase was also up-regulated in the ES and MS stages for valine, leucine and isoleucine biosynthesis, which are precursors of aroma compounds such as 2-methyl-propanal, 2-methyl-butanal and 3-methyl-butanal [9, 49]. Genes involved in the catabolism of aromatic amino acids tyrosine (*melO*), phenylalanine (*AO090038000057*) and tryptophan (*AO090038000578*) were induced, where the related catabolites can contribute to flavor formation [9]. By contrast, multiple amino acids were converted to energy for fast growth in TS2, hindering the production of flavor precursors necessary in soy sauce flavor formation.

Here, different survival strategies are utilized by RD2 and TS2: TS2 exploits glucose efficiently and burns extra amino acids to fuel glycolysis and TCA cycle for mycelium growth, making it a superior strain in survival competition, whereas RD2 sacrifices mycelium growth for elevated hydrolytic enzyme production and emphasizes metabolic pathways of flavor precursor formation, making it a favorable strain in koji fermentation. Upon the efficiency of nutrient utilization, TS2 is a superior strain with a higher adaptability to the koji fermentation environment. Since fast growth via induced glycolytic catabolism has extensive impact on both hydrolytic enzyme production and flavor precursor formation, the control of glycolysis and TCA cycle at the transcriptional level represents a possible solution. The gene *AZF1* has been reported to extensively induce the glycolytic catabolic pathways in *S. cerevisiae* [37]. Here, homologs of *AZF1* (*AO090003001179*) was found up-regulated in TS2 in the ME stage, which may be related to the regulation of glycolysis and TCA cycle. Also, a set of up-regulated TFs of mycelium growth that may influence glycolysis and TCA cycle in TS2 were identified here: homologs of *ASG1* (*AO090003001246*, *AO090009000029*), homologs of *ECM22* (*AO090023000416*, *AO090010000097*) and *UPC2* (*AO090009000133*, *AO090010000546*), which have redundant role in up-regulation of filamentous growth in *S. cerevisiae* [27, 46]. Of these, *AO090023000416* was up-regulated at about fivefold in TS2 in the three fermentation stages, possibly supporting the overall faster growth but repressing the production of hydrolytic enzymes and flavor precursor formation. The exact relationship between mycelium growth and fermentation performance with certain involvement of regulators need to be tested and discussed in future studies.

### Regulation on the formation of other flavor precursors

RD2 showed a stronger metabolic capacity of fatty acid, which are important flavor precursors in soy sauce [7]. For instance, triglyceride lipases (*AO090012000352*, *AO090001000143*, *AO090701000542*), fatty acid synthase (*fas1*, *AO090011000046*), aldehyde dehydrogenase (*AO090023000467*), and alcohol dehydrogenases (*AO090003000261*, *AO090003001407*) were up-regulated in RD2. The up-regulation of these enzymes largely induced the accumulation of fatty acids, which can be further catabolized by up-regulated aldehyde dehydrogenases and alcohol dehydrogenases to important aroma compounds, such as (*E*)-2-octenal, 1-octen-3-one, 5-methyl-3-heptanone, 3-octanol, and 1-octen-3-ol, which have been detected in mature koji [9, 38, 49]. Three up-regulated TFs (*AO090005000336*, *AO090001000029*, *AO090023000362*) with a predicted role in fatty acid catabolism may be responsible for the induction of aldehyde dehydrogenase and alcohol dehydrogenase. Moreover, this process is believed to be related to the sporulation of *A. oryzae* [16]. Here, better conidiation observed in RD2 probably supports the enhanced fatty acid metabolism.

Secondary metabolite isoprenoids can contribute to the overall aroma of fermented soy sauce [13]. Tetraterpenoid known as carotenoid can be readily catalyzed to various odor-active 2-butanone derivatives detected in soy sauce koji [9, 30, 38]. Here, acetyl-CoA acetyltransferase (*AO090103000406*) and isopentenyl-diphosphate Delta-isomerase (*AO090023000500*) that can induce the elongation of isoprenoid backbone and phytoene dehydrogenase (*AO090020000158*) used in the biosynthesis of carotenoid were up-regulated in RD2. The corresponding regulator hydroxymethylglutaryl-CoA reductase (*AO090701000640*) [45] was up-regulated in RD2, which may be responsible for the induced isoprenoid metabolism.

## Conclusions

In this study, we applied comparative genomics to examine genome variants in the genome of TS2 compared to RD2, and performed comparative transcriptomics to investigate differential gene expression patterns between the two strains in three stages of soy sauce koji fermentation. Our results showed that the phenotypic characteristics of TS2 could be explained by the genotypic defects observed—SNVs were found in genes related to the production of hydrolytic enzymes and formation of flavor precursors, indicating the importance of massive and intact reserve of hydrolytic enzymes and flavor precursors in ideal koji fermentation. The mutated gene loci may therefore represent molecular markers for koji fermentation performance. Transcriptomic analysis has confirmed the advantages of RD2 in hydrolytic enzyme production and flavor precursor formation, and rationalized corresponding regulation mechanisms involving fermentation parameters such as pH and conidiation of *A. oryzae* via identification of a set of TFs. The growth advantage of TS2 probably results from enhanced central carbon metabolism and amino acid catabolism, which in turn extensively lower the fermentation performance, suggesting the necessity of manipulating these metabolic pathways for desirable fermentation. By integrated comparative genomes and transcriptomes, a list of potential molecular markers related to desirable koji fermentation was identified here. This allows future evaluation of the fermentation capacity of *A. oryzae* strains by monitoring the gene structure, production level and activity of targeted proteins. Our study has therefore provided insights into targeted strain maintenance and strain improvement for a better fermentation process and soy sauce quality.

## Electronic supplementary material

Below is the link to the electronic supplementary material.

Supplementary material 1 (PDF 1220 kb)
