# Supplementary material for: Genomic and transcriptomic comparison of Aspergillus oryzae strains: a case study in soy sauce koji fermentation
Source: J Ind Microbiol Biotechnol. 2018 Jul 5;45(9):839–53. doi: 10.1007/s10295-018-2059-8 (PMC6105210; doi:10.1007/s10295-018-2059-8)
Supplement: Supplementary file 1 — Supplementary material 1 (PDF 1220 kb) [file 10295_2018_2059_MOESM1_ESM.pdf]

Supplementary material

Journal of Industry Microbiology and Biotechnology

Genomic and transcriptomic comparison of *Aspergillus oryzae* strains: A case study in soy sauce koji fermentation

Yiyi Zhong[a], Xi Lu[b], Lei Xing[a], Shiu Woon Allen Ho[c], Hoi Shan Kwan[a b #]

[a]School of Life Sciences, The Chinese University of Hong Kong, Hong Kong SAR, China

[b]Food Research Centre, The Chinese University of Hong Kong, Hong Kong SAR, China

[c]Lee Kum Kee International Holdings Limited, Hong Kong SAR, China

[#]Corresponding author:

Tel: 852-39436251

Fax: 852-39431146

Email: hoishankwan@cuhk.edu.hk

Table S1 Summary of transcriptome sequencing

		RD2_ME_1	RD2_ME_2	RD2_ES_1	RD2_ES_2	RD2_MS_1	RD2_MS_2
total reads		28,472,942	26,552,312	28,230,124	28,205,532	29,053,906	28,665,698
reads mapped to genome	#	26,014,816	24,265,361	25,799,666	25,747,189	26,585,685	26,191,184
	%	91.37%	91.39%	91.39%	91.28%	91.50%	91.37%
reads uniquely mapped to genome	#	25,642,167	23,985,103	25,559,005	25,481,493	26,255,807	25,686,732
	%	90.06%	90.33%	90.54%	90.34%	90.37%	89.61%
unmapped reads	#	2,458,126	2,286,951	2,430,458	2,458,343	2,468,221	2,474,514
	%	8.63%	8.61%	8.61%	8.72%	8.50%	8.63%
		TS2_ME_1	TS2_ME_2	TS2_ES_1	TS2_ES_2	TS2_MS_1	TS2_MS_2
total reads		27,332,472	27,769,506	31,820,890	27,373,578	31,352,208	29,079,946
reads mapped to genome	#	24,967,114	25,445,245	29,150,236	25,144,884	28,634,173	26,605,715
	%	91.35%	91.63%	91.61%	91.86%	91.33%	91.49%
reads uniquely mapped to genome	#	24,739,260	25,170,405	28,853,000	24,952,608	28,342,393	26,385,683
	%	90.51%	90.64%	90.67%	91.16%	90.40%	90.73%
unmapped reads	#	2,365,358	2,324,261	2,670,654	2,228,694	2,718,035	2,474,231
	%	8.65%	8.37%	8.39%	8.14%	8.67%	8.51%

Table S2 Primers of six selected genes used in real-time RT-PCR

Target	Putative function	Primers	Sequence (5'-3')
AO090120000026	Xylanase G2	xynG2_F	CATACAGCGTGCAATGGTCC
		xynG2_R	CAGCTAGGTAGCCGTTTCCG
AO090010000746	Glucoamylase	glaA_F	GCTCTTTACAGTTCTGCCGC
		glaA_R	ATGCAGCGTAGGTTTGGACG
AO090011000052	Leucine aminopeptidase	lapA_F	CGGGAGCTGATGACGATGGA
		lapA_R	CCTCTCCGGCATAGAAGTGG
AO090001000135	Neutral protease II	nptII_F	GACTAGCACCTGCCATGCTC
		nptII_R	AGTTGAGGTTGACAGCGTTGG
AO090012000706	Carboxypeptidase C	cpC_F	TGATTTCTCGGGACACGACG
		cpC_R	GTCTCCGGCTGGTAATAGGG
AO090011000235	Tripeptidyl-peptidase	tppA_F	AGAACGAACAGGAAATCCCCG
		tppA_R	AGAGTCACCGGAGGAGAAGA
AO090009000281 [2]	Beta-tubulin	btuA_F	CCAAGAACATGATGGCTGCT
		btuA_R	CTTGAAGAGCTCCTGGATGG

Table S3 Single nucleotide polymorphism (SNP) calling and insertion-deletion (indel) calling in TS2

Chr	Position	Reference (RD2)	Variance (TS2)	Quality[a]	RD[b]	Effect	Gene	Function
1	265874	G	A	39.52	5	Missense	AO090009000093	Geranylgeranyl pyrophosphate synthase
1	265883	G	A	96.14	6	Missense	AO090009000093	Geranylgeranyl pyrophosphate synthase
1	265921	C	A	92.14	6	Missense	AO090009000093	Geranylgeranyl pyrophosphate synthase
1	1464045	T	C	84.27	8	Missense	AO090009000555	C6 transcription factor
1	1602307	C	T	71.52	4	Missense	AO090009000600	Ketopantoate reductase
1	2694047	G	A	104.14	6	Missense	AO090005001409	Predicted protein
1	2694053	G	A	71.27	6	Missense	AO090005001409	Predicted protein
1	2946981	G	T	23.00	6	Missense	AO090005001296	Predicted transcriptional factor involved in carbohydrate
1	3593344	A	G	78.52	4	Missense	AO090005001067	Predicted protein
2	611783	G	T	24.99	3	Nonsense	AO090001000249	Fatty acyl-CoA synthase
2	665722	C	G	26.98	3	Missense	AO090001000266	Beta-glucosidase-related glycosidases
2	2994159	G	T	67.52	4	Missense	AO090003000329	Acyl transferase
2	3591915	G	T	61.27	5	Nonsense	AO090003000561	Predicted monooxygenase
2	3591919	A	C	54.27	5	Missense	AO090003000561	Predicted monooxygenase
2	4638013	A	T	80.27	5	Missense	eIF-5A	Translation initiation factor 5A
2	4638050	G	C	80.27	5	Missense	eIF-5A	Translation initiation factor 5A
2	4638064	A	C	58.52	4	Missense	eIF-5A	Translation initiation factor 5A
2	5973046	G	A	38.98	3	Missense	AO090003001444	Predicted protein
2	5973056	T	C	32.98	3	Missense	AO090003001444	Predicted protein
2	5973073	A	G	59.52	4	Missense	AO090003001444	Predicted protein
2	5973889	G	C	90.27	6	Missense	AO090003001444	Predicted protein
3	100935	C	T	53.52	4	Missense	AO090023000039	Transmembrane arginine transporter
3	984636	C	G	31.98	4	Missense	AO090023000382	Carboxypeptidase C
3	1016960	C	G	58.52	4	Missense	AO090023000396	Predicted protein
3	1040800	A	G	124.07	7	Missense	AO090023000405	MFS transporter
3	1130846	A	G	48.52	5	Missense	AO090023000444	Putative polyketide synthase
3	3107321	A	G	54.27	6	Missense	AO090026000734	Transmembrane amino acid transporter

3	3479001	A	T	20.02	5	Missense	AO090026000589	Predicted protein
3	4150441	C	T	43.97	3	Missense	AO090026000340	Protein related to DNA repair
3	4389287	C	A	29.98	3	Missense	AO090026000257	UDP-N-acetylmuramate dehydrogenase
4	936971	A	T	36.98	3	Missense	AO090012000379	Predicted protein
4	936982	A	C	43.97	3	Missense	AO090012000379	Predicted protein
4	937001	C	T	60.97	3	Missense	AO090012000379	Predicted protein
4	937029	T	A	78.27	5	Missense	AO090012000379	Predicted protein
4	963878	T	A	34.98	3	Missense	AO090012000392	Predicted protein
4	2927389	A	G	25.99	5	Missense	AO090701001170	Predicted protein
4	3310499	C	T	59.52	4	Missense	AO090102000448	Predicted protein
4	3310507	C	T	41.52	4	Missense	AO090102000448	Predicted protein
4	3656315	C	G	82.14	10	Missense	AO090102000326	Predicted protein
4	3657498	G	A	71.27	5	Missense	AO090102000326	Predicted protein
4	4237116	C	G	90.14	7	Missense	AO090102000117	Predicted protein
4	4881480	T	G	99.14	6	Missense	AO090166000123	Predicted protein
5	19526	T	C	82.14	8	Missense	AO090701000899	Predicted carbohydrate hydrolase activity
5	150257	T	C	65.97	4	Missense	AO090701000843	Predicted protein
5	836641	T	A	30.98	3	Missense	AO090701000573	Predicted protein
5	839676	C	T	93.27	8	Missense	AO090701000572	Glycosyl transferase family 8 protein
5	919311	G	C	96.14	6	Missense	AO090701000541	NACHT and WD40 domain protein
5	1059454	A	G	75.27	5	Missense	AO090701000493	Transcription factor involved in arginine biosynthesis
5	2578602	T	C	79.27	7	Missense	AO090124000043	Predicted protein
5	4104607	T	G	59.97	3	Missense	CYP5109A1	Cytochrome P450 monooxygenase
5	4271084	G	A	58.97	3	Missense	AO090113000091	Alpha-1,3-glucanase
5	4291566	C	T	112.07	7	Missense	AO090113000102	Predicted protein
6	278018	C	T	108.04	10	Missense	AO090020000610	Predicted protein
6	998373	A	G	80.27	5	Missense	AO090020000331	Pyridoxamine phosphate oxidase
6	2292874	T	C	30.98	8	Missense	AO090038000511	ABC drug exporter AtrF
6	2426950	C	T	93.27	5	Missense	AO090038000465	Prediced glutathione S-transferase

6	2544209	A	G	60.52	4	Missense	AO090038000421	Predicted protein
6	3401514	A	G	81.27	7	Missense	AO090038000105	Predicted protein
6	3736196	T	C	116.07	7	Missense	AO090138000190	Predicted protein
7	228694	T	C	41.97	3	Missense	AO090011000078	Predicted protein
7	857396	C	A	64.52	4	Missense	AO090011000336	Cysteine synthase B
8	305983	G	A	40.97	3	Missense	AO090701000995	Predicted protein
8	305985	C	A	39.98	3	Missense	AO090701000995	Predicted protein
8	305986	C	A	31.98	3	Missense	AO090701000995	Predicted protein
8	305987	A	T	38.98	3	Missense	AO090701000995	Predicted protein
8	305990	T	C	43.97	3	Missense	AO090701000995	Predicted protein
8	306019	C	T	91.27	7	Missense	AO090701000995	Predicted protein
8	316432	A	G	23.00	3	Missense	AO090103000369	Predicted protein
8	316443	G	A	23.00	3	Missense	AO090103000369	Predicted protein
8	814732	T	A	47.52	6	Missense	AO090103000176	Predicted protein
8	819338	A	C	62.52	5	Missense	AO090103000173	Predicted monooxygenase
8	819351	C	T	52.52	5	Missense	AO090103000173	Predicted monooxygenase
8	821551	G	C	41.97	3	Missense	AO090103000172	Lipase 2 precursor
8	919564	C	T	44.97	4	Missense	AO090103000131	Putive activator of maltose utilization
8	2048133	A	G	73.52	4	Missense	AO090010000501	Predicted protein
8	2117105	T	C	33.98	3	Missense	AO090010000476	Predicted protein
8	2673146	T	A	56.97	3	Missense	AO090010000781	Predicted protein
8	2673154	C	T	69.52	4	Missense	AO090010000781	Predicted protein
8	2673155	G	T	65.52	4	Missense	AO090010000781	Predicted protein
8	3126136	A	T	101.14	6	Missense	AO090010000108	Fatty acid synthase subunit beta
8	3134397	A	G	36.98	4	Missense	AO090010000107	3-oxoacyl-[acy-carrier-protein] synthase
2	595416	CA	C	111.59	6	Frameshift	AO090001000243	Predicted UDP-N-acetylmuramate dehydrogenase
2	4334639	AGG	AG	51.47	7	Frameshift	AO090003000832	MFS superfamily permease involved in amino acid
3	4458152	TGG	TG	36.47	6	Frameshift	AO090026000236	Predicted transcription regulator
5	564	AAGAGA	AAGAGAGAGA	214.53	7	Frameshift	AO090701000907	Predicted tripeptidyl-peptidase

5	4291655	GAAA	GAA	128.53	9	Frameshift	AO090113000102	Predicted protein
7	518225	ACCC	ACC	43.72	6	Frameshift	AO090011000199	Predicted protein
7	1230053	CGGG	CGG	66.72	5	Frameshift	AO090011000493	Structural maintenance of chromosome protein 4
8	1752092	GTTT	GTTTT	151.47	10	Frameshift	AO090010000625	Predicted protein
8	1936383	AGTATTCGTACCACGGTAT	AGTAT	205.72	7	Frameshift	CYP52K1	Cytochrome P450 monooxygenase

[a]Quality score generated by samtools

[b]Read depth of SNPs or indels

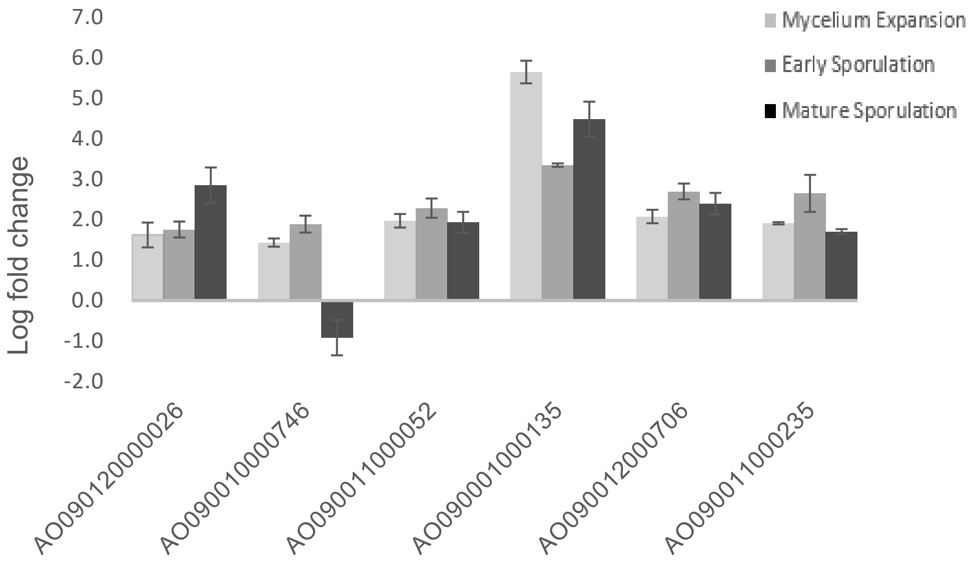

Fig. S1 Gene expression fold change of six selected genes in RD2 compared to TS2. Data is presented as mean ± standard deviation.

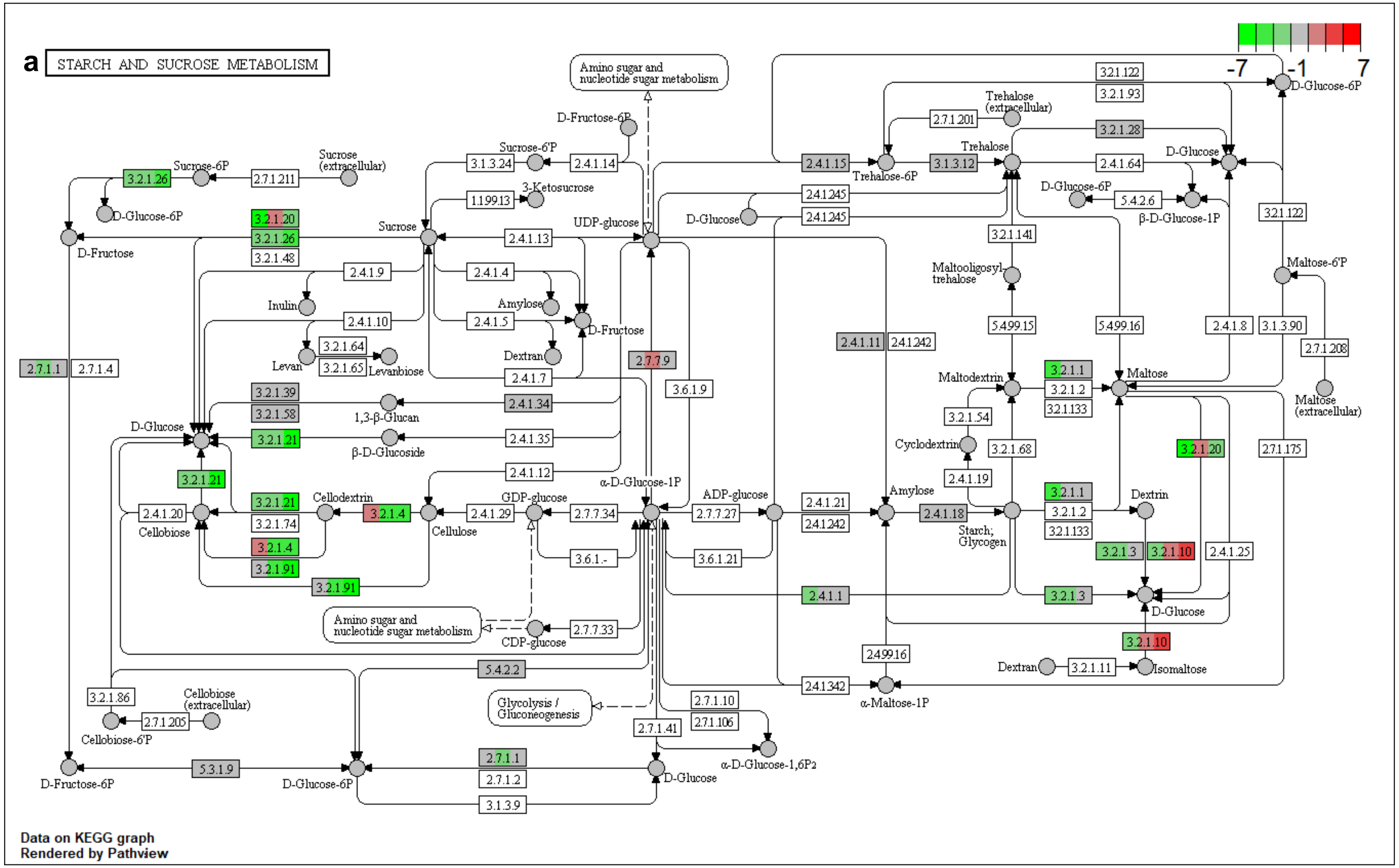

2.4.1.1, 3.2.1.1 alpha amylase

2.7.1.1 hexokinase

2.7.7.9 UDP-glucose pyrophosphorylase

3.2.1.3 glucoamlyase

3.2.1.4 endoglucanase

3.2.1.10 oligo-1,6-glucosidase

3.2.1.20 alpha-glucosidase

3.2.1.21 beta-glucosidase

3.2.1.26 beta-fructofuranosidase

3.2.1.91 cellulose 1,4-beta-cellobiosidase

Fig. S2 Differentially expressed genes (DEGs) of RD2 and TS2 mapped in starch and sucrose metabolism (**a**) and galactose metabolism (**b**) generated by Pathview [1]. The numbers in the boxes in-between compounds are Enzyme Commission numbers (EC numbers) of corresponding enzymes. Except the blank box representing enzymes unidentified in *A. oryzae* genome, each box is divided into three color blocks representing the mycelium expansion, the early sporulation and the mature sporulation stage in koji-making process (in a left-to-right order). The block highlighted in green or red means high expression level in RD2 or TS2, respectively, according to the legend in the upper right. The block in gray means no differential expression between two strains.

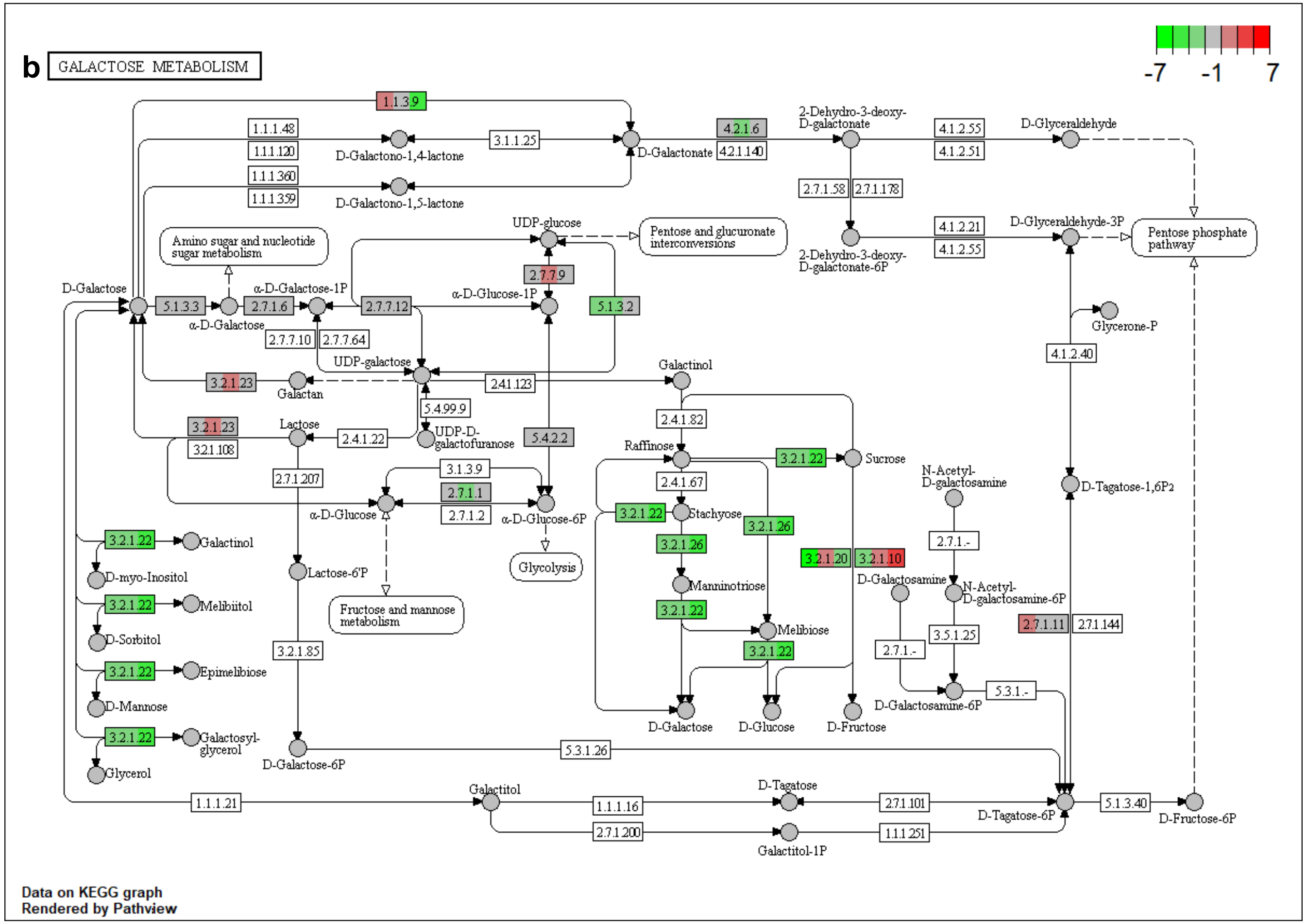

1.1.3.9 galactose oxidase
2.7.1.1 hexokinase
2.7.7.9 UDP-glucose pyrophosphorylase
2.7.1.11 6-phosphofructokinase 1
3.2.1.10 oligo-1,6-glucosidase
3.2.1.20 alpha-glucosidase
3.2.1.22 alpha-galactosidase
3.2.1.23 beta-galactosidase
3.2.1.26 beta-fructofuranosidase
4.2.1.6 galactonate dehydratase
5.3.1.2 UDP-glucose 4-epimerase

Fig. S2 (cont'd).

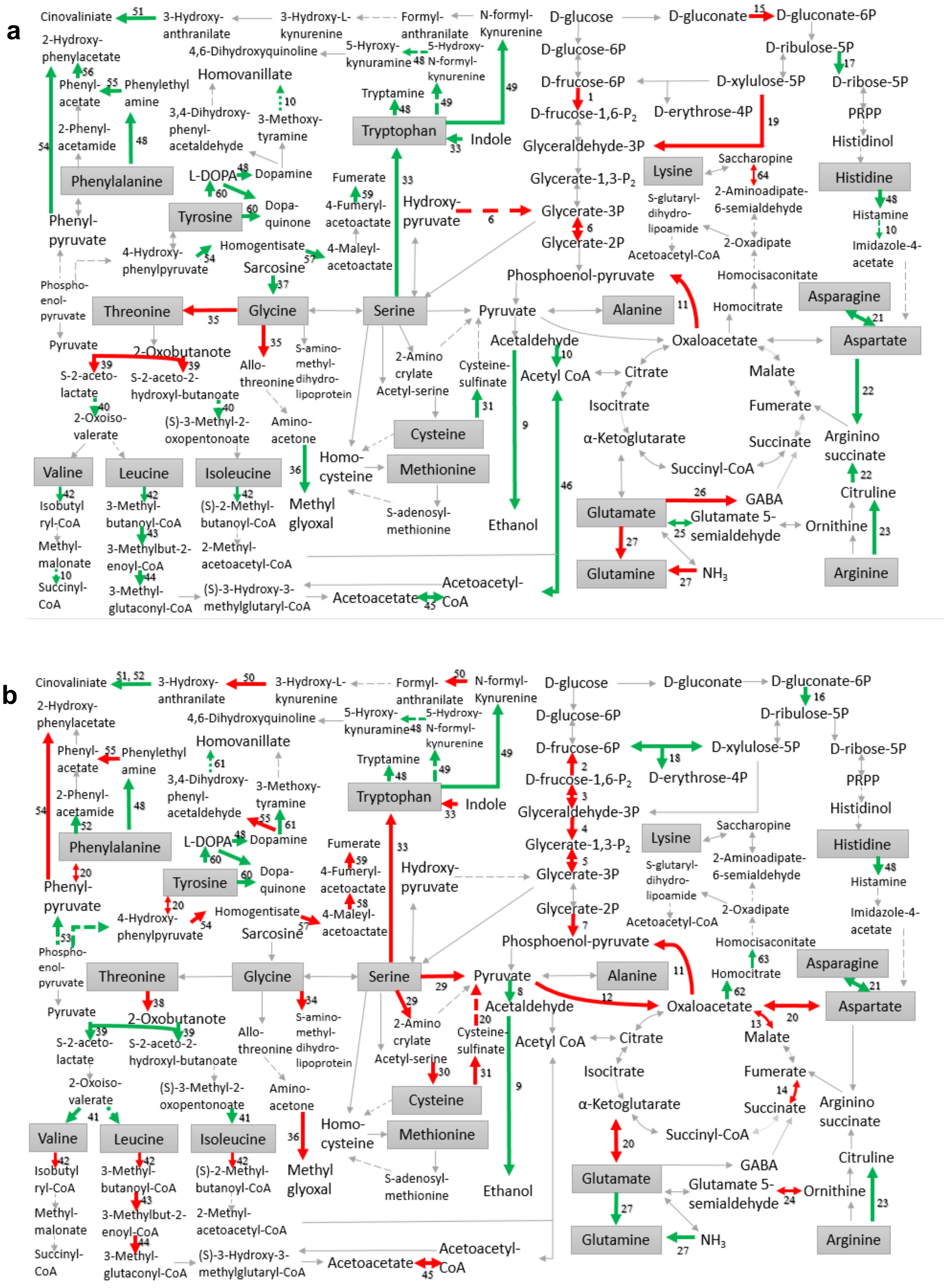
a
Cinovaliniate
3-Hydroxy-anthranilate
3-Hydroxy-L-kynurenine
Formyl-anthranilate
N-formyl-Kynurenine
D-glucose
D-gluconate
D-gluconate-6P
2-Hydroxy-phenylacetate
4,6-Dihydroxyquinoline
5-Hyroxy-kynuramine
5-Hydroxy-N-formyl-kynurenine
D-glucose-6P
D-ribulose-5P
Phenyl-acetate
Phenylethyl amine
Homovanillate
D-frucose-6P
D-xylulose-5P
D-ribose-5P
2-Phenyl-acetamide
3,4-Dihydroxy-phenyl-acetaldehyde
3-Methoxy-tyramine
Tryptamine
Tryptophan
Indole
D-frucose-1,6-P$_2$
D-erythrose-4P
PRPP
Histidinol
Glyceraldehyde-3P
Phenylalanine
L-DOPA
Dopamine
Fumerate
Glycerate-1,3-P$_2$
Lysine
Saccharopine
Histidine
Tyrosine
Dopa-quinone
4-Fumeryl-acetoacetate
Hydroxy-pyruvate
Glycerate-3P
S-glutaryl-dihydro-lipoamide
2-Aminoadipate-6-semialdehyde
Histamine
Phenyl-pyruvate
4-Hydroxy-phenylpyruvate
Homogentisate
4-Maleyl-acetoactate
Sarcosine
Glycerate-2P
Acetoacetyl-CoA
2-Oxadipate
Imidazole-4-acetate
Phospho-enol-pyruvate
Phosphoenol-pyruvate
Homocisaconitate
Asparagine
Threonine
Glycine
Serine
Pyruvate
Alanine
Homocitrate
Aspartate
2-Oxobutanote
S-amino-methyl-dihydro-lipoprotein
Allo-threonine
2-Amino crylate
Acetyl-serine
Cysteine-sulfinate
Acetaldehyde
Oxaloacetate
Acetyl CoA
Citrate
Malate
S-2-aceto-lactate
S-2-aceto-2-hydroxyl-butanoate
Isocitrate
Fumerate
2-Oxoiso-valerate
(S)-3-Methyl-2-oxopentonoate
Amino-acetone
Cysteine
α-Ketoglutarate
Succinate
Arginino succinate
Homo-cysteine
Methionine
Succinyl-CoA
Valine
Leucine
Isoleucine
Citruline
Isobutyl ryl-CoA
3-Methyl-butanoyl-CoA
(S)-2-Methyl-butanoyl-CoA
Methyl glyoxal
S-adenosyl-methionine
Ethanol
Glutamate
GABA
Glutamate 5-semialdehyde
Ornithine
Methyl-malonate
3-Methylbut-2-enoyl-CoA
2-Methyl-acetoacetyl-CoA
Glutamine
NH$_3$
Arginine
Succinyl-CoA
3-Methyl-glutaconyl-CoA
(S)-3-Hydroxy-3-methylglutaryl-CoA
Acetoacetate
b
Cinovaliniate
3-Hydroxy-anthranilate
3-Hydroxy-L-kynurenine
Formyl-anthranilate
N-formyl-Kynurenine
D-glucose
D-gluconate
D-gluconate-6P
2-Hydroxy-phenylacetate
4,6-Dihydroxyquinoline
5-Hyroxy-kynuramine
5-Hydroxy-N-formyl-kynurenine
D-glucose-6P
D-ribulose-5P
Phenyl-acetate
Phenylethyl amine
Homovanillate
D-frucose-6P
D-xylulose-5P
D-ribose-5P
Tryptamine
2-Phenyl-acetamide
3,4-Dihydroxy-phenyl-acetaldehyde
3-Methoxy-tyramine
Tryptophan
Indole
D-frucose-1,6-P$_2$
D-erythrose-4P
PRPP
Histidinol
Glyceraldehyde-3P
Phenylalanine
L-DOPA
Dopamine
Fumerate
Glycerate-1,3-P$_2$
Lysine
Saccharopine
Histidine
Tyrosine
Dopa-quinone
4-Fumeryl-acetoacetate
Hydroxy-pyruvate
Glycerate-3P
S-glutaryl-dihydro-lipoamide
2-Aminoadipate-6-semialdehyde
Histamine
Phenyl-pyruvate
4-Hydroxy-phenylpyruvate
Homogentisate
4-Maleyl-acetoactate
Sarcosine
Glycerate-2P
Acetoacetyl-CoA
2-Oxadipate
Imidazole-4-acetate
Phospho-enol-pyruvate
Phosphoenol-pyruvate
Homocisaconitate
Homocitrate
Asparagine
Threonine
Glycine
Serine
Pyruvate
Alanine
Aspartate
Acetaldehyde
Oxaloacetate
2-Oxobutanote
S-amino-methyl-dihydro-lipoprotein
Allo-threonine
2-Amino crylate
Acetyl-serine
Cysteine-sulfinate
Acetyl CoA
Citrate
Malate
S-2-aceto-lactate
S-2-aceto-2-hydroxyl-butanate
Isocitrate
Fumerate
2-Oxoiso-valerate
(S)-3-Methyl-2-oxopentonoate
Amino-acetone
Cysteine
α-Ketoglutarate
Succinate
Arginino succinate
Homo-cysteine
Methionine
Succinyl-CoA
Valine
Leucine
Isoleucine
Citruline
Isobutyl ryl-CoA
3-Methyl-butanoyl-CoA
(S)-2-Methyl-butanoyl-CoA
Methyl glyoxal
S-adenosyl-methionine
Ethanol
GABA
Glutamate
Glutamate 5-semialdehyde
Ornithine
Methyl-malonate
3-Methylbut-2-enoyl-CoA
2-Methyl-acetoacetyl-CoA
Glutamine
NH$_3$
Arginine
Succinyl-CoA
3-Methyl-glutaconyl-CoA
(S)-3-Hydroxy-3-methylglutaryl-CoA
Acetoacetate

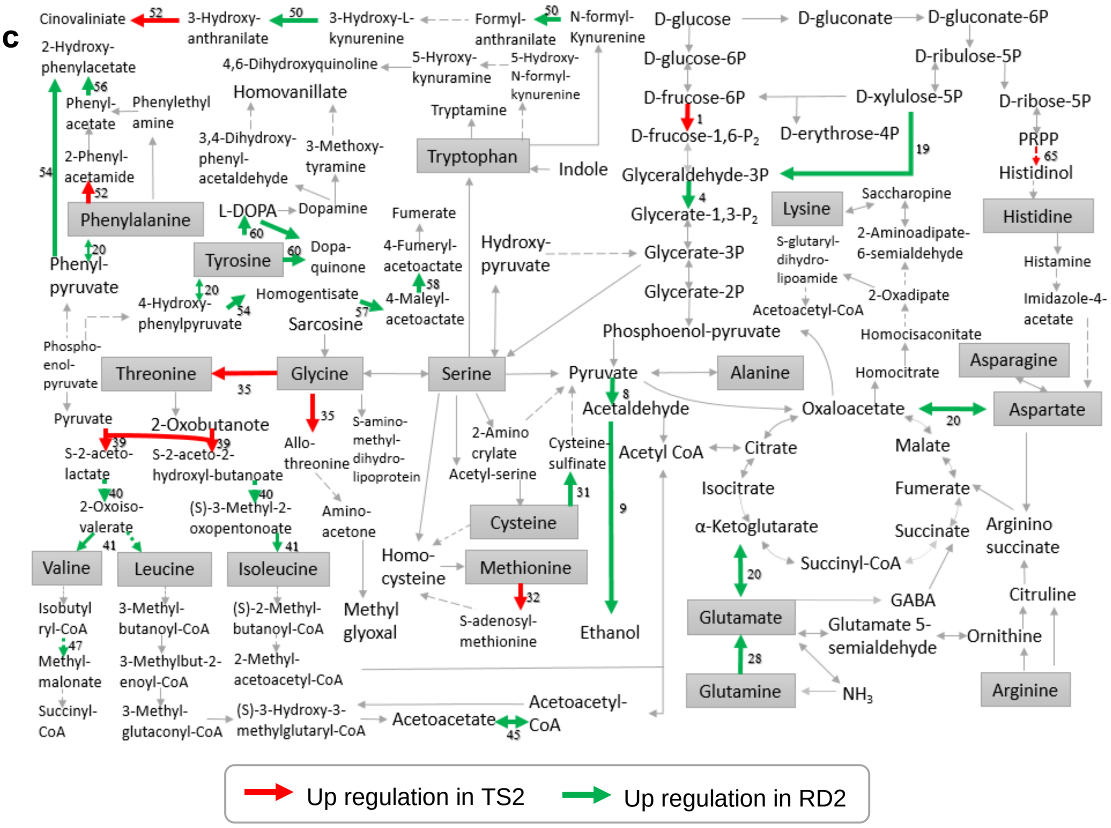

Fig. S3 Modified KEGG pathways showing differentially expressed genes in the mycelium expansion (**a**), early sporulation (**b**) and mature sporulation (**c**) stages that were mapped on the central carbon metabolism and related amino acid metabolism. Red and green arrows indicate upregulated reactions in TS2 and RD2, respectively; dashed arrows indicate more than one step of reaction involved. DEG numbers correspond to the enzymes listed in Table 2. Abbreviation: P, phosphate; PRPP, 5-Phospho-alpha-D-ribose 1-diphosphate; GABA, 4-aminobutanoate; L-DOPA, 3,4-dihydroxy-L-phenylalanine

References

1. Luo W, Brouwer C (2013) Pathview: an R/Biocondutor package for pathway-based data integration and visualization. Bioinformatics 29:1830-1831

2. Tominaga M, Lee YH, Hayashi R, Suzuki Y, Yamada O, Sakamoto K, Gotoh K, Akita O (2006) Molecular analysis of an inactive aflatoxin biosynthesis gene cluster in *Aspergillus oryzae* RIB strains. Appl Environ Microbiol 72:484-490
